# Assessment of knowledge and perceptions of health professionals towards One Health in Somaliland

**DOI:** 10.3389/fvets.2026.1753866

**Published:** 2026-06-03

**Authors:** Yusuf Mohamed Yusuf, Abdirizak Mohamud Yusuf, Abdimajid Said Siad, Abdullahi Ahmed Tahlil, Saed Ahmed Ismail, Abdi Ibrahim Mohammed, Mohamed Ismael Iman

**Affiliations:** 1 Department of Public Health, Ministry of Health Development, Hargeisa, Somalia; 2 Falkiye Research Institute, Mogadishu, Somalia; 3 Department of Public Health, Federal Ministry of Health, Mogadishu, Somalia; 4 National Institute of Health, Federal Ministry of Health, Mogadishu, Somalia; 5 Hargeisa Group Hospital, Hargeisa, Somalia; 6 Faculty of Medicine, Al Hayat Medical University, Mogadishu, Somalia

**Keywords:** health professionals, knowledge, One Health, perceptions, Somaliland

## Abstract

**Introduction:**

The One Health (OH) approach emphasizes the interconnectedness of human, animal, plant, and environmental health within shared ecosystems, aiming to foster multisectoral collaboration to address complex health challenges such as zoonoses, antimicrobial resistance, and climate change. Despite its global recognition, the operationalization of OH in Somaliland remains limited, hindered by systemic challenges, inadequate awareness, and insufficient intersectoral coordination. This study assesses the knowledge and perceptions of health professionals regarding OH in Somaliland to inform policy and capacity-building strategies.

**Methods:**

A descriptive cross-sectional survey was conducted from June to August 2024 across six regions of Somaliland, involving 422 licensed health professionals from diverse disciplines, including physicians, veterinarians, nurses, environmental health specialists, and public health officers. Data were collected using a structured, pretested questionnaire translated into Somali, encompassing socio-demographic variables, knowledge, and perceptions of OH. Data analysis was used SPSS Version 25 and involved descriptive statistics, chi-square tests, and multivariate logistic regression to identify factors associated with knowledge and perception levels.

**Results:**

The findings revealed that 71.8% of participants demonstrated poor knowledge of OH, with only 28.2% exhibiting adequate understanding. Over half (56.6%) held negative perceptions of OH, citing limited awareness, lack of formal training, and absence of institutional support as contributing factors. Multivariate analysis indicated that higher educational attainment, age above 39 years, and being a physician significantly increased the likelihood of possessing good knowledge (AOR = 30.71, *p* < 0.001) and positive perceptions. Conversely, health professionals with less experience or in non-medical disciplines demonstrated lower awareness and less favorable attitudes toward OH.

**Conclusion:**

The study underscores substantial gaps in knowledge and perception of the OH approach among Somaliland’s health professionals, impeding effective multisectoral engagement. To advance OH implementation, targeted capacity-building initiatives, curriculum integration, policy development, and enhanced intersectoral collaboration are imperative. Strengthening awareness and professional training will be critical to fostering a resilient health system capable of addressing zoonotic and environmental health threats in Somaliland.

## Introduction

One Health (OH) is an integrated, multisectoral strategy that acknowledges the vital interdependence between human, animal, plant, and environmental health within a shared ecosystem (1, 2). This forward-looking approach promotes collaboration across disciplines including public health, veterinary medicine, agriculture, and environmental science to tackle today’s most pressing global health challenges, such as zoonotic diseases, antimicrobial resistance (AMR), climate change, and food insecurity (1, 3). This method brings together diverse sectors, disciplines, and communities from all levels of society to enhance health and address environmental concerns. It also prioritizes access to clean water, power, and air, as well as safe and nutritious food, while combating climate change and fostering sustainable development (4). By fostering holistic and inclusive solutions, OH aligns with the Sustainable Development Goals (SDGs) and global health security agendas, championing a united front among the World Health Organization (WHO), Food and Agriculture Organization (FAO), United Nations Environment Programme (UNEP), and World Organization for Animal Health (WOAH) in combating cross-species disease transmission and ecological degradation (4, 5).

Zoonotic diseases alone account for over 60% of all known infectious diseases and approximately 75% of emerging pathogens, inflicting an estimated 2.5 billion illnesses and 2.7 million deaths annually worldwide (6–10). The worldwide status of One Health is marked by its growing acknowledgement and practical application, particularly in the context of global health security and the Sustainable Development Goals. This approach, which recognizes the interdependence of human, animal, and ecosystem health, has the potential to transform healthcare by fostering interdisciplinary cooperation and communication (11). However, its operationalization at the global level is hindered by challenges such as institutional proliferation, fragmentation, and competition for resources (12). In fact, there is a need for a transdisciplinary approach that involves collaboration among multiple disciplines and the development of new methods and tools for research and implementation (11).

The frequency of devastating outbreaks such as Ebola, COVID-19, and Rift Valley Fever has surged in recent years, exposing vulnerabilities in siloed health systems and highlighting the urgent need for integrated, cross-sectoral responses (13). In Africa, zoonoses have led to over 28,000 reported infections and more than 1,182 deaths in the past decade, with outbreaks increasing by 63% in Sub-Saharan Africa alone and accounting for one-third of public health emergencies in the region over the last 20 years (14, 15). These figures are not just statistics; they underscore the escalating impact of fragmented health governance. Despite the growing urgency, the implementation of OH remains fragmented, particularly in fragile and conflict-affected settings where institutional capacity is limited and health systems are chronically underfunded (16). In Somalia, the OH approach was formally introduced in 2013, yet its adoption has been slow and uneven due to persistent instability, weak governance structures, and a reliance on externally funded, short-term interventions (16). While promising initiatives such as the Somali One Health Centre (SOHC) have emerged, they remain nascent and largely disconnected from national health planning and policy frameworks (17).

Somaliland, a self-declared republic in north-western Somalia reflects many of these same systemic challenges. With a predominantly pastoralist population and high human-animal contact, the region is especially vulnerable to zoonotic outbreaks. Yet efforts to institutionalize OH are stymied by limited intersectoral collaboration, lack of formal training for health professionals, and an absence of integrated policy frameworks (18). Funding constraints, weak technical capacity, and a fragmented health infrastructure further restrict the scale and sustainability of OH initiatives (16). In contrast, neighboring countries such as Ethiopia and Kenya have demonstrated the impact of strong political will, university-government partnerships, and community-led engagement in embedding OH principles into national policy and practice (19, 20). What makes this context especially concerning is the absence of baseline data on the knowledge, attitudes, and perceptions of frontline health professionals, those most critical to operationalizing OH on the ground. Without understanding how OH is perceived among healthcare providers, veterinarians, environmental officers, and other key sectors, strategic planning and implementation are bound to falter.

To our knowledge, this is the first study assessing the awareness and attitudes of health professionals toward the One Health approach in Somaliland. The findings offer an invaluable snapshot of current readiness levels, gaps in education and intersectoral coordination, and opportunities for systemic reform. By leveraging the One Health Systems Framework which emphasizes coordination, governance, and capacity-building this study not only evaluates Somaliland’s preparedness for OH adoption but also identifies locally relevant strategies to strengthen collaboration at the human-animal-environment interface. In doing so, this research adds to the global OH evidence base while shining a spotlight on a region too often overlooked in international discourse. More importantly, it seeks to empower local actors’ policymakers, academic institutions, and development partners with actionable insights to build resilient health systems capable of withstanding future zoonotic and environmental threats.

## Methods and materials

### Study design and setting

This study was designed as a descriptive cross-sectional study to provide a comprehensive assessment of the current knowledge and perceptions of health professionals regarding the One Health (OH) concept within Somaliland. The cross-sectional approach was chosen because it allows for the collection of data at a single point in time, offering a snapshot of the prevailing attitudes and understanding among diverse health sector professionals. This design is particularly suitable for identifying gaps in knowledge, misconceptions, and attitudes that could influence the implementation of One Health initiatives. Data collection was conducted over a three-month period from June to August 2024 to ensure temporal consistency and to facilitate logistical planning. The study was conducted in Somaliland, a semi-autonomous region located on the southern coast of the Gulf of Aden. The region shares borders with Djibouti, Ethiopia, and Somalia, with Hargeisa serving as its capital and administrative hub. Hargeisa hosts key government ministries including health, livestock, and environment as well as numerous healthcare facilities, both public and private. The city’s strategic importance and diversity of health facilities made it an ideal location for capturing a representative sample of health professionals practicing across various sectors. To ensure broader geographical representation, the study included participants from six different regions within Somaliland, thus capturing a wide range of perspectives and practices related to the One Health approach.

### Source and study population

The study targeted licensed health professionals actively engaged in human health, animal health, and environmental health sectors across Somaliland. The population included practitioners from multiple disciplines, such as physicians, veterinarians, nurses, midwives, public health officers, health administrators, environmental health specialists, nutritionists, laboratory technicians, pharmacists, dentists, and optometrists. These professionals worked within a variety of healthcare settings, including government health facilities, non-governmental organizations (NGOs), private clinics, and research institutions. The inclusion of a diverse range of health professionals was essential for capturing the multifaceted nature of the One Health concept, which emphasizes the interconnectedness of human, animal, and environmental health. The study aimed to encompass professionals from all relevant sectors to better understand the level of integration and awareness across disciplines that are critical for successful One Health implementation.

### Inclusion criteria and exclusion criteria

Participants eligible for inclusion in this study were licensed health professionals actively practicing in Somaliland and willing to participate voluntarily during the study period. Conversely, individuals who did not hold valid health professional licenses, those not currently practicing in Somaliland, or those who declined to participate or refused to consent to the study were excluded from the study to maintain data integrity and relevance.

### Sample size determination

The sample size was calculated based on the single proportion formula, with the assumption that the proportion of health professionals possessing adequate knowledge or positive perceptions of One Health was unknown and no previous study has been conducted in Somaliland regarding this topic, warranting a proportion estimate of 50%. Using a 95% confidence level (corresponding to a Z-score of 1.96) and a margin of error of 0.05. The calculation was performed as follows: $n^{\prime }=\frac{Z^{2}\times p\times (1-p)}{e^{2}}=\frac{{\left(1.96\right)}^{2}\times 0.5\times (1-0.5)}{{\left(0.05\right)}^{2}}=384$ .

To accommodate potential non-responders and incomplete questionnaires, an additional 10% was added: total = 384 + 38 = 422. So, the total required sample size was 422. This sample size was deemed sufficient to achieve the desired statistical power and precision for the study.

### Sampling technique and procedure

**Figure 1:**
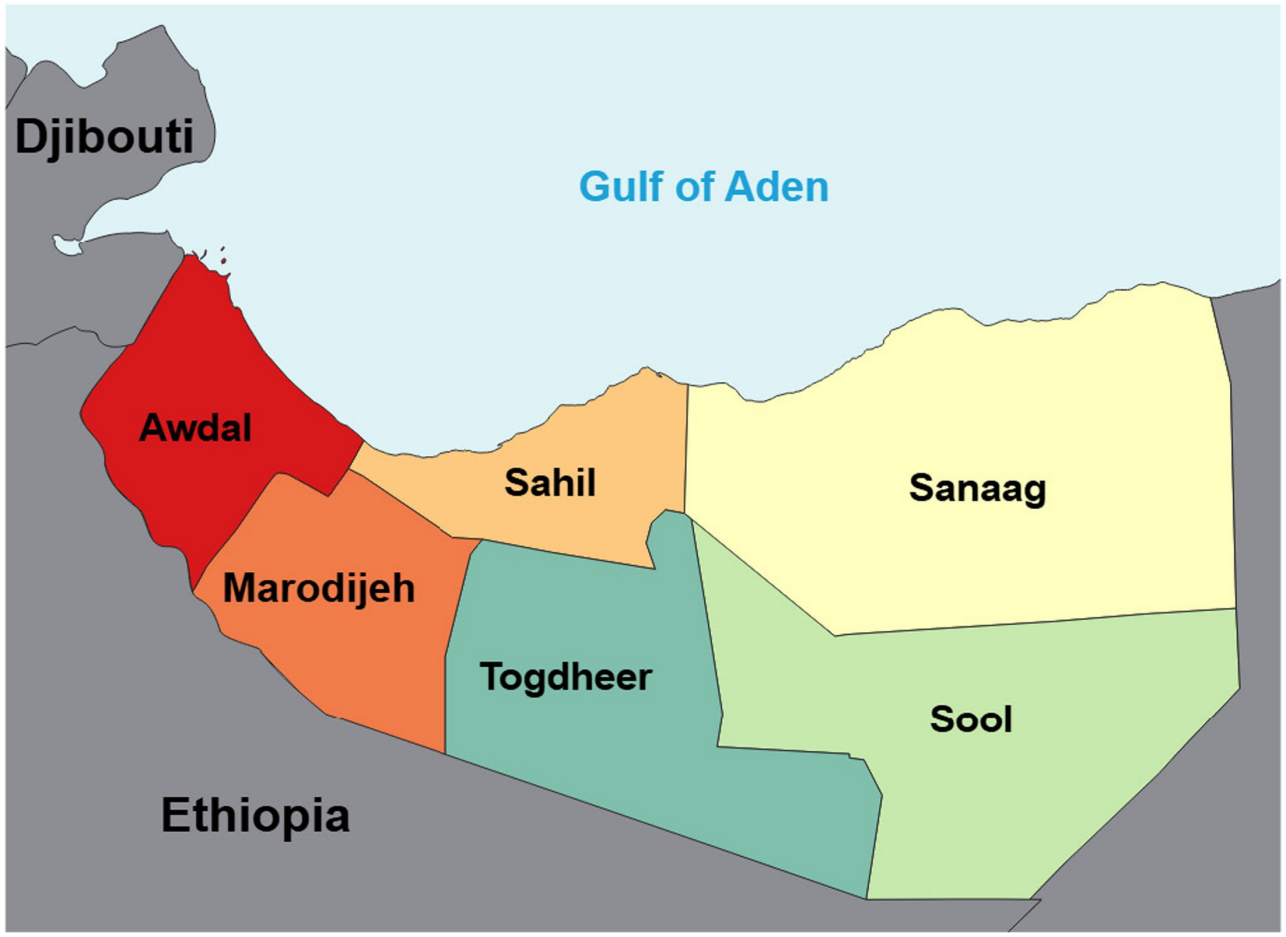
Map showing the geographical locations of the study in Somaliland.

A stratified simple random sampling technique was employed. Healthcare facilities within Hargeisa and surrounding regions were stratified based on their type such as hospitals, outpatient clinics, and veterinary clinics (Figure 1). A comprehensive list of health professionals within these strata was compiled through official records obtained from relevant government ministries and institutional databases. From this list, participants within each stratum were selected systematically using a simple random sampling technique. This involved assigning a sequential number to each individual and selecting every *kth* individual, where *k* was determined based on the proportional size of each stratum relative to the total sample size. Prior to data collection, eligible participants were approached, briefed on the study objectives, and provided with detailed information about voluntary participation. Informed consent was obtained from all participants to uphold ethical standards and ensure voluntary engagement. This systematic and stratified approach was intended to produce a representative sample that accurately reflects the diverse professional backgrounds and geographic distributions of health professionals across Somaliland.

### Data collection instruments and techniques

Data collection was conducted using a structured, pretested questionnaire designed specifically for this study. The questionnaire was developed in English and translated into Somali and subsequently back-translated by a bilingual expert to enhance comprehension. The questionnaire was pilot-tested on 5% of the sample size, approximately 21 respondents, to assess its reliability, understandability, and cultural appropriateness. Based on feedback, necessary revisions were made to improve question clarity and reduce ambiguity. The final instrument comprised sections on socio-demographic information, knowledge about the One Health concept, and perceptions regarding its importance and implementation. The questionnaire was developed by the research team based on study objectives and relevant literature based on One Health in Somaliland, and was reviewed for clarity and content validity prior to data collection. Data were collected using structured, self-administered paper-based questionnaires, with trained data collectors present to provide clarification and support when needed. This approach minimized interviewer bias while ensuring participant understanding and completeness of responses. The questionnaire assessed both general understanding of the One Health concept and its key components, including the link between human, animal, and environmental health. It also included multiple items on perceived importance and implementation, capturing broader knowledge and practical relevance beyond single questions. In addition to that, the questionnaire used yes or no and multiple-choice items to assess knowledge, and Likert-scale questions to measure perceptions, enabling quantification of both concepts.

### Study variables

The dependent variables were knowledge and perceptions of health professionals concerning the One Health approach. Independent variables included socio-demographic factors such as age, sex, educational level, years of professional experience, professional discipline, and regional location. These variables were analyzed to identify potential associations and predictors influencing knowledge and perceptions.

### Data quality control

Data quality control and assurance are essential for ensuring the reliability and accuracy of research findings. This study implemented rigorous training for data collectors, pilot testing to refine the survey instrument, and continuous supervision to address challenges during data collection. A double-entry system was used during data entry, with regular checks and validation to minimize errors. Discrepancies identified during analysis was reviewed to maintain data consistency. These measures aimed to enhance the credibility of the study, ensuring meaningful conclusions on One Health knowledge, and attitudes in Somaliland.

### Data analysis

Data analysis was performed using IBM SPSS Statistics Version 25. Descriptive statistics such as frequencies, percentages, means, and standard deviations were used to summarize demographic characteristics and the distribution of knowledge and perception scores. Knowledge was scored based on correct responses to knowledge-based questions, with a threshold established to categorize participants into “adequate” or “inadequate” knowledge groups. Participants with scores above a predetermined cut-off (e.g., 75% for knowledge questions) were classified as having “adequate” knowledge, while similar thresholds were used for perceptions, categorized as “positive,” or “negative” based on cumulative scores on the Likert-scale items. Bivariate analyses involved cross-tabulation of socio-demographic variables against knowledge and perception categories. Chi-square tests were performed to identify any significant associations between demographic variables and Knowledge and perception levels. Variables with a *p*-value less than 0.25 in these analyses were considered for multivariate modeling to identify independent predictors. The multivariate analysis calculated adjusted odds ratios (AORs) with 95% confidence intervals (CIs). Model fit was assessed using the Hosmer-Lemeshow goodness-of-fit test, with a *p*-value >0.05 indicating good fit. Multicollinearity among independent variables was checked via Variance Inflation Factors (VIFs), ensuring VIFs below 10 to avoid multicollinearity bias.

## Results

### Socio-demographic characteristics of the study participants

Table 1 presents the demographic characteristics of the study participants. A total of 422 were approached to complete the survey, and because of the detailed pre-study information and consent process, 100% of those approached completed the survey. The participants’ ages ranged from 19 to over 67 years, with a mean age of 29 ± 6 years. The majority of participants, 242 (57.3%), were aged between 19 to 28 years. Most respondents, 252 (59.7%), were female. Regarding educational qualifications, most participants, 237 (56.2%), held a bachelor’s degree and represented a diverse range of health field professions, including nurses/midwives 135 (32.0%), followed by physicians 80 (19.0%), laboratory technicians 44 (10.4%), and public health practitioners (10.2%). A total of 117 (27.7%) had a diploma, a post-secondary qualification below a bachelor’s degree. The majority of participants, 147 (34.8%), resided in Maroodijeex, and 97 (23.0%) in Togdheer. Additionally, 284 (67.3%) participants had five or fewer years of professional experience, with an average of 5.2 ± 3.5 years. This data reflects a predominantly young, female workforce with diverse health-related roles and moderate professional experience. This diverse cohort provides a comprehensive overview of health professionals’ knowledge and perceptions of One Health within Somaliland.

**Table 1:** Socio-demographic characteristics of the study participants (*n* = 422).

Variables	Frequency (*n*)	Percentages
Age (in years)		
19–28	242	57.3
29–38	160	37.9
≥39	20	4.7
Sex		
Male	170	40.3
Female	252	59.7
Educational level		
Diploma	117	27.7
Bachelor	237	56.2
Master’s	66	15.6
Doctorate	2	0.5
Health field professions		
Physician	80	19.0
Veterinarian	23	5.5
Nurse/Midwife	135	32.0
Public health	43	10.2
Health administrator	14	3.3
Environmental health	16	3.8
Nutritionist	20	4.7
Lab technician	44	10.4
Pharmacist	14	3.3
Dentist	21	5.0
Optometrist	12	2.8
Region of residence		
Sool	27	6.4
Sanaag	40	9.5
Togdheer	97	23.0
Saaxil	46	10.9
Maroodijeex	147	34.8
Awdal	65	15.4
Years of experience		
≤5	284	67.3
>5	138	32.7

### Knowledge of health professionals towards One Health

Table 2 presents the health workers’ knowledge and familiarity with One Health (OH). The findings revealed that approximately two-thirds, 280 (66.4%) of respondents, had heard of One Health. Among those aware of One Health, the primary sources of information included courses 130 (46.4%) and television 51 (18.2%). On the other hand, lesser contributions came from research articles and social media, totaling 45 (16.1%) and 44 (15.7%), respectively. However, a significant proportion, 285 (67.5%), reported not having received any formal training on One Health. Among those who received training, online courses, 54 (39.4%), and graduate degrees, 47 (34.3%), were the most common avenues. Familiarity with One Health’s implications was low, with 190 (45.0%) of participants not being familiar and 121 (28.7%) being less familiar, while only 73 (17.3%) identified as familiar with One Health. These findings underscore the need for increased awareness and targeted educational interventions to enhance health professionals’ understanding of One Health in Somaliland.

**Table 2:** Knowledge of health professionals towards One Health (*n* = 422).

Variables	Frequency (*n*)	Percentage (%)
Ever heard of the concept of the One Health		
No	142	33.6
Yes	280	66.4
The main source of information on One Health (*n* = 280)		
Social media	44	15.7
Health professionals	10	3.6
Course	130	46.4
Television	51	18.2
Research articles	45	16.1
Received any formal training on the One Health		
No	285	67.5
Yes	137	32.5
Place of training (*n* = 137)		
Continuing vocational education	10	7.3
Online course training	54	39.4
Symposium/conference	20	14.6
University (undergraduate degree)	11	8.0
University (graduate degree)	47	34.3
Familiar with the concept of One Health and its implications		
Not familiar	190	45.0
Less familiar	121	28.7
Familiar	73	17.3
More familiar	38	9.0

### Perception of health professionals towards One Health

Table 3 presents the perceptions of health professionals towards One Health. A majority, 284 (67.3%), of participants acknowledge the significance of One Health in addressing global health challenges. Regarding zoonotic diseases, 164 (38.9%) perceive them as posing a moderate threat, while 98 (23.2%) view them as a high threat to public health. A substantial proportion, 272 (64.5%), believe that climate change adversely impacts both human and animal health. Over half of the respondents, 240 (56.9%), agree that integrating One Health principles into healthcare practices can enhance patient outcomes. Furthermore, 263 (62.3%) concur that promoting the One Health approach can aid in preventing disease outbreaks across human and animal populations. However, perceptions regarding the effectiveness of government policies in advancing One Health initiatives are less optimistic, with nearly half, 209 (49.5%), viewing policies as ineffective, and only 60 (14.2%) regarding them as more effective. A substantial majority, 299 (70.9%), agree that education and awareness campaigns are vital for fostering recognition of One Health among healthcare professionals and the public.

**Table 3:** Perception of health professionals towards One Health (*n* = 422).

Variables	Frequency (*n*)	Percentage (%)
I believe that the One Health is important for addressing global health challenges		
No	138	32.7
Yes	284	67.3
I think that zoonotic diseases pose a threat to public health		
Low	160	37.9
Moderate	164	38.9
High	98	23.2
I believe that climate change impacts human and animal health		
No	150	35.5
Yes	272	64.5
I believe that incorporating One Health principles into healthcare practice can improve patient outcomes		
No	182	43.1
Yes	240	56.9
I believe that promoting the One Health approach can help prevent disease outbreaks in humans and animals		
No	159	37.7
Yes	263	62.3
I believe that government policies are effective in promoting One Health initiatives and research		
Not effective	209	49.5
Less effective	153	36.3
More effective	60	14.2
I believe that education and awareness campaigns can promote the importance of the One Health among healthcare professionals and the public		
No	123	29.1
Yes	299	70.9
I believe the One Health approach is essential for preventing and controlling zoonotic diseases.		
Strongly disagree	66	15.6
Disagree	92	21.8
Neutral	84	19.9
Agree	103	24.4
Strongly agree	77	18.2
I am confident in the collaborative efforts between medical, veterinary, and environmental professionals within the One Health framework in Somaliland.		
Strongly disagree	104	24.6
Disagree	174	41.2
Neutral	50	11.8
Agree	55	13.0
Strongly agree	39	9.2
I feel that the resources allocated to the One Health program in Somaliland are adequate to achieve its objectives		
Strongly disagree	108	25.6
Disagree	185	43.8
Neutral	51	12.1
Agree	47	11.1
Strongly agree	31	7.3
I believe training and education on the One Health approach are well-integrated into the professional development of health professionals		
Strongly disagree	120	28.4
Disagree	152	36.0
Neutral	42	10.0
Agree	61	14.5
Strongly agree	47	11.1
I believe the One Health program’s strategies are practical and applicable in real-world settings		
Strongly disagree	64	15.2
Disagree	66	15.6
Neutral	95	22.5
Agree	138	32.7
Strongly Agree	59	14.0

Perceptions about the role of One Health in disease prevention are mixed: 103 (24.4%) agree and 77 (18.2%) strongly agree that it is essential for controlling zoonoses. Confidence in collaborative efforts among medical, veterinary, and environmental professionals is relatively low, with 174 (41.2%) disagreeing and 104 (24.6%) strongly disagreeing. Similarly, resource allocation appears inadequate, with 185 (43.8%) disagreeing and 108 (25.6%) strongly disagreeing that those resources are sufficient for achieving One Health’s objectives. In terms of professional development, 152 (36%) of participants do not believed that training and education on One Health are well integrated. Despite this, over a third, 138 (32.7%), agree that the strategies of the One Health program are practical and applicable in real-world settings, with 59 (14.0%) strongly agreeing. These findings highlight the urgent need for improved policy frameworks, increased funding, enhanced training initiatives, and strengthened collaboration to fully realize the potential of One Health.

### Factors associated with the level of knowledge and One Health among health professionals

Table 4 presents the multivariate logistic regression analysis of factors associated with the level of knowledge on One Health among health professionals in Somaliland. Several variables showed significant associations with good knowledge after adjusting for potential confounders. Age was significantly associated with knowledge levels towards One Health. Professionals aged 39 years and above demonstrated 5.87 times higher odds (AOR = 5.87, 95% CI: 1.39–24.71, *p* = 0.016) of possessing good One Health knowledge compared to younger professionals (19–28 years). This may reflect accumulated experience, longer exposure to multidisciplinary health concepts, or greater participation in advanced training over time. Educational level demonstrated a strong positive association with knowledge levels. Participants with a Bachelor’s degree had 7.08 times higher odds (AOR = 7.08, 95% CI: 2.81–17.87, *p* < 0.001), while those with master’s or doctoral qualifications had 30.71 times higher odds (AOR = 30.71, 95% CI: 10.23–92.16, *p* < 0.001) of good One Health knowledge compared to diploma holders. Higher education likely provides broader exposure to interdisciplinary health concepts, research, and training that align with One Health principles. Professional discipline significantly influenced One Health knowledge. Health administrators (AOR = 0.24; 95% CI: 0.07–0.86; *p* = 0.029), environmental health professionals (AOR = 0.28; 95% CI: 0.09–0.90; *p* = 0.033), and dentists (AOR = 0.33; 95% CI: 0.12–0.91; *p* = 0.032) were significantly less likely to have good knowledge of One Health compared to physicians. Physicians may receive more integrated training on zoonotic diseases, antimicrobial resistance, and human-animal-environment interactions during medical education.

**Table 4:** Multivariate logistic regression of demographic factors associated with knowledge levels of One Health among health professionals in Somaliland (*n* = 422).

Variables	Knowledge Level		COR (95%CI)	AOR (95%CI)	*p* value
	Poor	Good			
Age (in years)					
19–28	190	52	1	1	
29–38	109	51	1.04 (0.64–1.70)	0.88 (0.47–1.62)	0.669
≥ 39	4	16	6.81 (2.91–15.94)	5.87 (1.39–24.71)	0.016*
Sex					
Male	107	63	2.06 (1.34–3.17)	0.95 (0.54–1.67)	0.852
Female	196	56	1		
Educational level					
Diploma	111	6	1		
Bachelor	168	69	7.60 (3.19–18.10)	7.08 (2.81–17.87)	<0.001*
Master’s & doctorate	24	44	33.92 (12.98–88.61)	30.71 (10.23–92.16)	<0.001*
Health field professions					
Physician	45	35	1	1	
Veterinarian	9	14	1.30 (0.48–3.53)	1.83 (0.63–5.32)	0.266
Nurse/Midwife	109	26	0.27 (0.15–0.48)	0.52 (0.26–1.04)	0.063
Public health	23	20	0.72 (0.34–1.53)	0.68 (0.30–1.53)	0.350
Health administrator	12	2	0.23 (0.07–0.79)	0.24 (0.07–0.86)	0.029*
Environmental health	10	6	0.34 (0.11–1.04)	0.28 (0.09–0.90)	0.033*
Nutritionist	19	1	0.24 (0.08–0.70)	0.32 (0.10–1.03)	0.056
Lab technician	39	5	0.29 (0.14–0.64)	0.48 (0.21–1.12)	0.089
Pharmacist	12	2	0.16 (0.04–0.60)	0.28 (0.06–1.25)	0.095
Dentist	17	4	0.43 (0.16–1.13)	0.33 (0.12–0.91)	0.032*
Optometrist	8	4	0.57 (0.17–1.93)	0.65 (0.18–2.34)	0.506

* Significant value at *p* < 0.05.

### Factors associated with level of perception and One Health among health professionals

The multivariate logistic regression analysis identified several statistically significant factors associated with a positive perception of One Health among health professionals in Somaliland, based on Adjusted Odds Ratios (AOR). Educational level was a strong predictor of positive perception. Professionals with a bachelor’s degree had 4.64 times higher odds (AOR = 4.64, 95% CI: 2.54–8.49, *p* < 0.001) of having a positive perception compared to those with a diploma. Similarly, those with a master’s or doctorate degree exhibited 7.22 times higher odds (AOR = 7.22, 95% CI: 3.09–16.90, *p* < 0.001) of a favorable perception. This suggests that advanced education enhances understanding of One Health principles, likely due to greater exposure to interdisciplinary training, research, and public health concepts. Health profession also significantly influenced perception levels. Health administrators (AOR = 0.24, 95% CI: 0.07–0.86, *p* = 0.029), environmental health professionals (AOR = 0.28, 95% CI: 0.09–0.90, *p* = 0.033), and dentists (AOR = 0.33, 95% CI: 0.12–0.91, *p* = 0.032) were significantly less likely to have a positive perception of One Health. Physicians may have greater exposure to zoonotic diseases and human-animal-environment interactions, making them more receptive to One Health. Professional experience showed a significant association with perception, as individuals with more than 5 years of experience had a 44% lower likelihood of reporting a positive perception compared to those with five or fewer years (AOR = 0.56, 95% CI: 0.32–0.97, *p* = 0.040). This suggests that increased experience may positively influence perceptions toward One Health (Table 5).

**Table 5:** Multivariate logistic regression of demographic factors associated with perception levels of One Health among health professionals in Somaliland (*n* = 422).

Variables	Perception Level		COR (95%CI)	AOR (95%CI)	*P* value
	Negative	Positive			
Age (in years)					
19–28	141	101	1	1	
29–38	91	69	1.06 (0.71–1.59)	0.63 (0.36–1.09)	0.097
≥39	7	13	2.59 (0.99–6.73)	1.43 (0.43–4.73)	0.557
Sex					
Male	76	94	2.27 (1.52–3.37)	1.44 (0.88–2.36)	0.152
Female	163	89	1	1	
Educational level					
Diploma	97	20	1	1	
Bachelor	115	122	5.15 (2.99–8.87)	4.64 (2.54–8.49)	<0.001*
Master’s & doctorate	27	41	7.37 (3.72–14.59)	7.22 (3.09–16.90)	<0.001*
Health field professions					
Physician	29	51	1	1	
Veterinarian	7	16	1.30 (0.48–3.53)	1.83 (0.63–5.32)	0.266
Nurse/Midwife	92	43	0.27 (0.15–0.48)	0.52 (0.26–1.04)	0.063
Public health	19	24	0.72 (0.34–1.53)	0.68 (0.30–1.53)	0.350
Health administrator	10	4	0.23 (0.07–0.79)	0.24 (0.07–0.86)	0.029*
Environmental health	10	6	0.34 (0.11–1.04)	0.28 (0.09–0.90)	0.033P
Nutritionist	14	6	0.24 (0.08–0.70)	0.32 (0.10–1.03)	0.056
Lab technician	29	15	0.29 (0.14–0.64)	0.48 (0.21–1.12)	0.089
Pharmacist	11	3	0.16 (0.04–0.60)	0.28 (0.06–1.25)	0.095
Dentist	12	9	0.43 (0.16–1.13)	0.33 (0.12–0.91)	0.032*
Optometrist	6	6	0.57 (0.17–1.93)	0.65 (0.18–2.34)	0.506
Years of experience					
≤5	154	130	1	1	
>5	85	53	0.74 (0.49–1.12)	0.56 (0.32–0.97)	0.040*

* Significant value at *p* < 0.05.

### Overall knowledge and perception level of health professionals towards One Health

**Figure 2:**
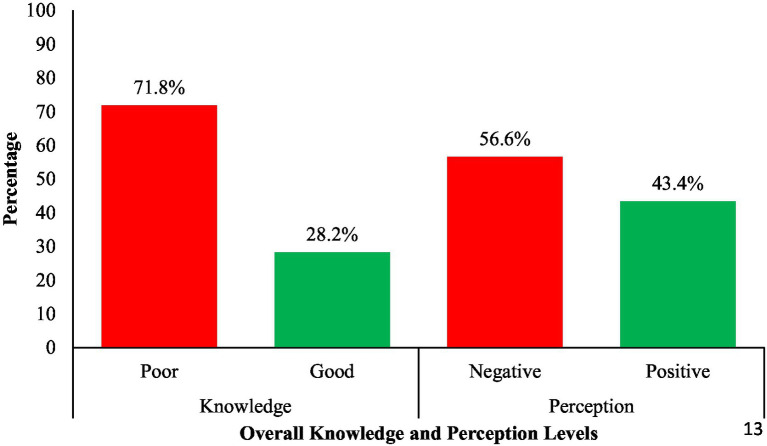
Overall, knowledge and perception levels of health professionals towards One Health (*n* = 422).

The findings reveal that a significant majority of participants, 303 (71.8%), demonstrated poor knowledge of One Health, while only 119 (28.2%) exhibited a good level of awareness. This suggests a critical need for enhanced education and training on One Health principles among health professionals. This study also shows that the majority of the participants, 239 (56.6%), revealed a negative perception of One Health. Conversely, 183 (43.4%) of the participants demonstrated a positive perception of the One Health. Negative perceptions may stem from lack of awareness, misconceptions, or insufficient advocacy about the benefits of One Health (Figure 2).

## Discussion

This study assessed the knowledge and perceptions of health professionals towards the One Health approach in Somaliland. The findings reveal that a substantial proportion of participants (71.8%) demonstrated poor knowledge of the One Health concept. This indicates a significant knowledge gap among health professionals in the country, which could impede the effective implementation of integrated health strategies targeting human, animal, and environmental health.

This study is a similar with findings from other study conducted in Bhutan reported that only 63% of health professionals demonstrated adequate knowledge of One Health (21). This aligns closely with findings from Türkiye, where 63.5% of participants reported never having heard of OH (22). Similarly, research from China revealed that just 40% of professionals showed a favorable perception of One Health, citing lack of integration between sectors as a primary barrier (23). A multicentre cross-sectional study across 26 African countries found that while 88.2% of Central African students demonstrated adequate OH knowledge, students from North Africa exhibited the lowest scores (64.1%) (24). The even lower awareness in Somaliland may reflect the country’s particular challenges, including limited structured OH education and formal mechanisms for cross-sectoral collaboration (25). The low level of awareness in Somaliland may be attributed to the absence of formal One Health education in the health curricula, limited intersectoral collaboration, and inadequate governmental or institutional advocacy for One Health practices. These findings suggest an urgent need for capacity building, targeted training programs, and institutional policy development to promote the One Health approach at the national level.

The findings of this study reveal that a significant proportion of health professionals in Somaliland (56.6%) exhibited a negative perception of the One Health. This trend indicates a considerable gap in awareness, integration, or acceptance of the One Health concept among health sector stakeholders in the region. The negative perception observed in our findings is higher than that reported in studies conducted in Nigeria, where only 69.7% of health professionals were found to have a negative perception of One Health (26). Similarly, a study in Bangladesh reported that 62.5% of professionals had a negative perception of One Health, particularly those engaged in zoonotic disease management and collaborative disease surveillance (27). In contrast, the perception level in Somaliland is consistent with findings from Nepal, where approximately 56.4.% of surveyed professionals expressed limited understanding and a lack of enthusiasm for cross-sectoral collaboration under the One Health framework (28). The similarity may reflect systemic challenges common to fragile health systems, such as fragmented intersectoral collaboration, limited One Health education in university curricula, and minimal institutional support for integrated health efforts. The relatively low level of positive perception in Somaliland could be attributed to several factors. Firstly, the lack of formal education and training on the One Health approach in both medical and veterinary institutions may limit professionals’ understanding and appreciation of its importance. Secondly, the absence of a national One Health coordination mechanism or policy framework may result in minimal intersectoral collaboration, thereby reinforcing siloed approaches to health issues. Thirdly, there may be a general lack of awareness about zoonotic disease risks and the benefits of integrated health responses, particularly in rural and underserved areas.

Older professionals (≥39 years) demonstrated higher OH knowledge, likely due to accumulated experience and exposure to multidisciplinary health concepts, aligning with findings from Ethiopia where senior health workers showed better OH comprehension (1). Educational attainment was a strong predictor, with Bachelor’s and postgraduate holders exhibiting 7.08- and 30.71-times higher odds of good OH knowledge, respectively, compared to diploma holders. This mirrors trends in Uganda, where higher education integrates OH principles into curricula (24). Physicians outperformed health administrators, environmental health workers, and dentists in OH awareness, consistent with studies in Nigeria, where medical training emphasizes zoonotic diseases and antimicrobial resistance (29). Conversely, environmental health professionals in Somaliland lagged behind, contrasting with Rwanda, where targeted OH training improved their competency (30). Somaliland’s fragile health system, exacerbated by decades of conflict and underfunding, lacks structured OH education and cross-sectoral collaboration (17). Recent initiatives, such as the Capacitating One Health in Eastern and Southern Africa (COHESA) project, OH training in Borama and Mogadishu, show promise, with participants reporting improved knowledge in data-driven decision-making and community engagement (25). However, gaps persist, particularly among non-physician cadres, necessitating curriculum reforms and continuous professional development.

The findings of this study also shows that educational attainment significantly influences health professionals’ perceptions of One Health in Somaliland. Consistent with studies conducted in Nepal, higher educational levels were positively associated with better understanding and acceptance of One Health concepts, likely due to increased exposure to interdisciplinary training and public health education (28). Professionals holding a bachelor’s or higher degree demonstrated significantly higher odds of having a favorable perception, reinforcing the role of formal education in fostering awareness of zoonotic disease dynamics and intersectoral collaboration. Moreover, the variation in perception across health professions aligns with findings from Ethiopia, where physicians displayed more positive attitudes compared to other cadres, including environmental health professionals and administrators (31). This discrepancy may be attributed to the physicians’ frequent engagement with zoonoses and cross-disciplinary clinical challenges. Interestingly, professionals with more than 5 years of experience were less likely to exhibit positive perceptions, contrary to trends in Bhutan where experience enhanced understanding and support for One Health (21). This contrast suggests that experience alone may not suffice without continuous professional development in One Health concepts. This study is limited by its cross-sectional design, reliance on self-reported data, potential response bias, and limited generalizability, as well as scarce local evidence for comparison.

## Conclusion and recommendations

The study found that health professionals in Somaliland have limited knowledge and poor perceptions of the One Health program, with notable differences based on gender and profession. These gaps hinder effective implementation and highlight the need for targeted education, collaboration, and capacity building. To address these challenges, the study recommends providing targeted training, raising awareness, integrating One Health into academic curricula, enhancing interdisciplinary collaboration, developing supportive policies, and establishing systems for ongoing evaluation. For strengthening interdisciplinary collaboration, government support, CPD programs, and community engagement, along with continued research, will help bridge current gaps and ensure sustainable progress.

## Data Availability

The original contributions presented in the study are included in the article/supplementary material, further inquiries can be directed to the corresponding author.
